# A Novel Automated Participant-Recorded Dietary Data Collection Method Using Low-Cost Mobile Phones and Interactive Voice Response with Low-Literacy Women: A Validation Study in Rural Uganda

**DOI:** 10.1016/j.cdnut.2026.107678

**Published:** 2026-03-26

**Authors:** Lydia O’Meara, Kate Wellard, Joweria Nambooze, Patrick Ongora, Paula Dominguez-Salas, Elaine Ferguson

**Affiliations:** 1 Natural Resources Institute, University of Greenwich, London, UK; 2 Cornell Atkinson Center for Sustainability, Department of Public and Ecosystem Health, Cornell University, Ithaca, NY, United States; 3 Department of Nutritional Science and Dietetics, Kyambogo University, Kampala, Uganda; 4 Africa Innovations Institute, Kampala, Uganda; 5 Ichuli Institute, Lira, Uganda; 6 International Livestock Research Institute, Nairobi, Kenya; 7 Department of Epidemiology and Population Health, London School of Hygiene and Tropical Medicine, London, UK

**Keywords:** validation, inter-method accuracy and agreement, information and communication technology, dietary data collection and assessment, women of reproductive age, agriculture- and fisheries-dependent households, sub-Saharan Africa

## Abstract

**Background:**

Dietary data gaps limit the design of effective food policies and programs in low- and middle-income countries. Automated mobile phone-based tools offer an opportunity to fill data gaps using less resources compared with face-to-face methods, especially for high-frequency dietary quality monitoring in resource-constrained environments.

**Objectives:**

This study assessed the validity of automated participant-recorded dietary data via interactive voice response (IVR) on basic mobile phones to assess dietary quality among rural women in a sub-Saharan African context, against same-day gold standard observed weighed food records.

**Methods:**

Automated IVR collected list-based recalls of food groups consumed in the last 24 h using yes or no push-button response from 156 randomly selected women in rural Northern Uganda (wet season). Inter-method agreement was assessed by comparing mean women’s dietary diversity scores (WDDS) using Weighted Cohen’s kappa, Bland–Altman plots, and Wilcoxon signed-rank test. Agreement in percentage achieving minimum dietary diversity scores for women (MDD-W) and consumption of unhealthy foods and beverages was tested using Cohen’s kappa and McNemar’s test.

**Results:**

Most women (74.4%) completed the IVR, with completion associated with better network coverage and prior positive mobile phone experience. Compared with the weighed food records, agreement for the IVR was moderate for MDD-W (21.6% compared with 17.2%; *kappa* = 0.48), fair for mean WDDS (3.3 compared with 3.5; weighted *kappa* = 0.39), and moderate for unhealthy food (34.5% compared with 23.3%; *kappa* = 0.44) and beverage consumption (32.8% compared with 31.9%; *kappa* = 0.43).

**Conclusions:**

This study highlights the potential of collecting data from low-literate women in rural resource-scarce settings using IVR via basic mobile phones to estimate population-level MDD-W, WDDS, and percentage consuming unhealthy foods and beverages. Given the need for participant training, IVR may be best suited to high-frequency monitoring of sentinel groups across time. Further refinement of the IVR may improve food group reporting, reducing trade-offs between operational simplicity and accuracy. With appropriate adaptation, we expect this method is generalizable to other settings.

## Introduction

Low dietary quality and malnutrition remain leading global causes of morbidity and mortality [1], disproportionately affecting women and children in low- and middle-income countries (LMICs) [2]. Monitoring dietary quality, including adequacy, diversity, moderation, and overall balance, is essential for tracking progress toward achieving nutrition and food system goals [3,4]. Yet data gaps persist [5] due to logistical barriers to enumerator-administered methods, remoteness, poor infrastructure, mobility restrictions, and high resource needs [[6], [7], [8]], particularly during crises when dietary risks peak [[9], [10], [11]]. As climate, conflict, and infectious disease outbreaks (e.g., Ebola, Covid-19) increasingly disrupt food security and fieldwork [2], participant-recorded approaches show promise for filling data gaps and enabling cost-effective, real-time monitoring [[12], [13], [14], [15]].

Mobile phones, the most widespread fastest-growing 2-way communication channel in LMICs, are now owned by around 80% of women [16]. Though rural access remains lower than urban areas [17,18], phone coverage is expanding [16]. When there are mobility restrictions such as during the Covid-19 pandemic, virtual surveys were used to monitor diets, yet most phone-based methods are enumerator-dependent [9,[19], [20], [21]], whereas text- [14] and web-based methods [22] privilege higher-literate groups. Recent reviews indicate a scarcity of validated digital tools in LMICs [23], with limited assessment against gold standards [24]. Recent studies highlight the potential of participant-recorded phone-based dietary data collection in sub-Saharan Africa [7,14,25]. A study in Rwanda demonstrated the potential of using text messaging for assessing dietary diversity indicators among literate, higher-income adults [14]. In Kenya, a picture-based smartphone application was used to collect anthropometric and dietary data from nomadic women and children; although validity of the dietary component was not assessed [7]. In Ghana, an artificial intelligence-assisted photo-based application was validated for assessing nutrient intake among adolescent girls, but only in an urban context [25]. However, to date, interactive voice response (IVR) systems using basic mobile phones have not been validated for collecting data to assess dietary diversity indicators.

IVR systems using mobile phones have considerable potential for use in LMICs, enabling automated phone-based questionnaires where participants interact with high quality prerecorded scripts by talking into the phone or pressing buttons on the phone’s keypad [24,26]. The advantages of the automated design of IVR are that it reduces interviewer bias or error through consistent phrasing, pacing, and intonation of questions; it calls and records multiple participants simultaneously; it transcends low literacy levels; it can employ different languages; and process thousands of calls in a single day [26,27]. These IVR features encompassing convenience, simplicity, confidentiality, and cost savings offer advantages compared to conventional enumerator-administered methods [8]. In high-income countries, IVR has proven effective for health monitoring, such as chronic pain [27], and bowel symptoms [28]. In LMICs, IVR has been utilized to elicit information on health practices in Bangladesh [13], and household welfare indicators in Peru and Honduras [29].

The women’s dietary diversity score (WDDS) and minimum dietary diversity for women (MDD-W) are 2 standardized international indicators [30], validated as proxies for micronutrient adequacy among women of reproductive age (WRA) in low-resource settings [[31], [32], [33]]. MDD-W is a key sustainable development goal indicator [4] and it, along with WDDS, has been in wide global use over the last decade for monitoring dietary changes, as well as assessing drivers and effects of nutrition policies, programs, and interventions around women's dietary quality [11]. In recent years, there has also been a growing need to monitor unhealthy food consumption given the rising prevalence of diet-related noncommunicable diseases in LMICs [34]. To date, the list-based food group versions of collecting women's dietary diversity are enumerator administered [[35], [36], [37]]. Although large surveys have begun collecting women's dietary diversity at scale, these resource-intensive surveys are often only conducted every 4–10 y. To our knowledge, no validated automated participant-recorded IVR dietary data collection method currently exists for monitoring dietary quality of females in LMICs, especially among groups with low literacy.

This study builds on Phase I of an Innovative Methods and Metrics for Agriculture and Nutrition Actions (IMMANA) project which piloted same-day IVR dietary intake diaries among mother-child dyads in Eastern Uganda [38]. The pilot demonstrated high acceptability for IVR in rural, low-literacy settings but was constrained by the need to complete 3 calls per day, indicating lessons learned, which were applied in the current study. This included refinement of the IVR questionnaire, data quality platform settings, and implementation protocols, encompassing a simplified one-call format, intensive pre-testing, tailored support to promote equitable participation, and automated call-reconnect functions to mitigate network disruptions.

The objective of this study was to assess the feasibility and validity of automated participant-recorded dietary data collected via IVR on basic mobile phones to assess key international dietary diversity indicators among rural women in a sub-Saharan African context, against gold standard same day observed weighed food records (WFR). This study aimed to first refine a novel method of collecting women's dietary data using participant-recorded list-based recalls of food groups consumed in the previous 24 h administered via automated IVR with push button yes or no response on basic mobile phones with rural women in Northern Uganda. It then aimed to assess the validity of the IVR method for estimating MDD-W, WDDS, and the percentage of women consuming unhealthy foods and beverages by analyzing the inter-method agreement between the IVR and the WFR.

## Methods

### Study design

A cross-sectional study of WRA (18–49 y) with a biological child aged 12–23 mo (*n* = 156) was conducted during the wet season (August 29, 2022 to November 3, 2022) in Apac and Kwania districts, Northern Region, Uganda. The primary objective of the main study was to evaluate the use of mobile phones and wearable cameras for collecting data on diets, time use, and handwashing practices. The secondary objectives were to evaluate the use of accelerometers to evaluate energy expenditure, and to assess whether dietary micronutrient adequacy differed between women living close to or further away from large waterbodies. Only the feasibility and validity results of the mobile phone method are reported herein.

### Participants and sampling

This study was conducted in Lango, Northern Uganda, a sub-region 7–9 h drive from the capital city Kampala, with a low human capital development index and high malnutrition rates [39], reflecting significant disadvantage from historical conflict, unemployment, low education, and climate-related food insecurity. Apac and Kwania districts were purposively chosen for their proximity to large waterbodies (River Nile, Lake Kwania) supporting fisheries. Nationally, 41% of Ugandan females aged 10 y or older owned a mobile phone in 2024 compared with 46% of males, a 5-point gap [40]. In Northern Uganda, a recent UN assessment suggests that this gender gap in phone ownership is up to 25 points, linked with restrictive gender norms, affordability, low education, and rural isolation [18].

The target sample size (156 mother-child dyads) was determined based on 2 project objectives: *1*) validating a dietary data collection method, and *2*) examining the relationship between women's micronutrient adequacy and proximity to waterbodies. Previous dietary validation studies indicate that a minimum of 100 participants is required [[31], [32], [33]], consistent with the median sample size of 100–120 reported in systematic reviews of dietary [41] and diagnostic [42] validation studies. This sample size satisfies the minimum thresholds for assessing inter-method agreement using Bland–Altman plots [43], Cohen’s kappa to detect at 0.40 [44], and allows for 30% attrition [28].

**FIGURE 1:**
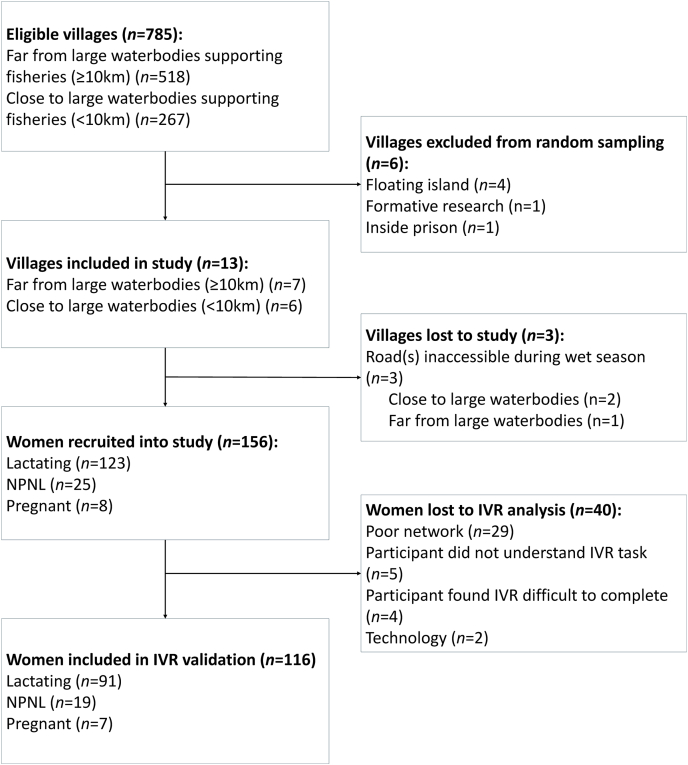
Flow diagram of participant recruitment. IVR, interactive voice response; NPNL, nonpregnant/nonlactating.

The sample size was also calculated to detect a 10% difference in the mean probability of adequacy (MPA) in nutrient intakes, for females living ≤10 km and >10 km from large waterbodies (α = 0.05, 80% power, SD = 0.2) [31], yielding 128 participants. This sample size was adjusted for clustering (design effect, 1.03942) [45] and 10% attrition, resulting in a minimum sample size of 149. Due to enumerator team configurations, the target was set at 156. Mother-child dyads were selected via 2-stage random sampling: 16 villages [8 near (<10 km), 8 far (>10 km) from waterbodies] [46] were randomly chosen from 785 eligible villages based on lists compiled by local government officials, with 3 later excluded due to severe flooding (2 close to waterbodies) (Figure 1). Villages located on floating islands or inside prisons or involved in the study’s formative research were also excluded. In each village, 12 eligible dyads (plus 3 reserves in selection order) were randomly selected from lists compiled by local leaders. Inclusion criteria included females aged 18–49 y, speaking Lleblango, residing in the village, having no discernible disability that impaired participation in the study, available to participate during the week of data collection, and being the biological mother of a child aged 12–23 mo. The inclusion criteria for the child were a singleton birth, had no discernible disability or illness affecting food consumption, currently eating solid foods, aged between 12 and 23 mo and available during data collection. If insufficient dyads were found in a village, replacements were randomly drawn from a nearby village. Sampling and random selection were calculated using R software “pwr,” “dplyr,” and “sample_n” [47].

### Data collection

Women's food and beverage consumption was assessed using 15 h observed WFRs, and list-based recalls of food groups consumed (yes/no response) in the previous 24 h using IVR. Data were collected over 3 consecutive days. Before data collection, a community sensitization workshop was held. On day 1, assessment of participant eligibility, and the collection of anthropometric data, and a structured questionnaire were completed at a central village location. On day 2, the enumerator shadowed the woman during her daily activities, including at the home, farm, and away from home, collecting WFR data covering that day’s intake. On day 3, the participant answered a phone call in the morning for the IVR list-based food group recall capturing dietary data for the same day as the WFR. In the afternoon of the third day, an enumerator visited the house to conduct a semistructured exit questionnaire. Data were collected on a rolling basis across weekdays and weekends to account for day-of-week effects at the population level [48,49], such as market days where consumption of foods consumed outside of the home can influence participant reporting [50]. For each participant, a different enumerator administered the WFR, IVR familiarization, and exit questionnaire. All staff had tertiary qualifications, were fluent in both English and Lleblango and were trained beforehand. Training took place over 3.5 wk (August 2–26, 2022). It comprised classroom training, role-play practice, and assessments with individualized feedback. Training also included 3 d of field practice in one of the target districts (Kwania).

### Observed WFR and anthropometry

Each participant’s food and beverage intakes were estimated using a 1 d WFR with dietary scales (±1g, Salter Disc Electronic Digital Scale Model 1036) [48]. A trained female enumerator accompanied the participant from 06:30 to 21:00, weighing all foods and beverages served and left over, including all foods consumed outside of the home. Recipe data were collected, including weighing raw ingredients, cooked food, and noting cooking methods. Foods or drinks consumed outside these hours were recorded using standard 24 h dietary recall methods for which the quantity of foods consumed were estimated using actual food, raw rice, or playdough models [51]. Cooked foods were adjusted using USDA yield factors [52], as per the Harvest Plus Ugandan Food Composition Table [53]. Duplicate anthropometric measurements (weight to 0.1 kg, height to 0.5 cm) were also taken (SECA weighing scales, Microtoise).

### Enumerator-administered questionnaires

Enumerators administered 2 structured questionnaires. The first covered socio-demographics, household wealth, food security, women's empowerment, nutrition/health knowledge, and sources and biodiversity of local foods. The second was an exit questionnaire, using Likert and categorical scales, assessing participant perceptions of the dietary data collection methods used in the study, phone ownership/use, and willingness for future mobile phone-based studies, with open-ended questions for qualitative feedback. The first questionnaire was collected using SurveyCTO software (Dobility Inc, v2022) on Android tablets, whereas the second was collected via paper-based methods.

### Novel automated IVR

#### Contextualization and pretesting

Key informant interviews, focus groups, and observational interactive pretesting with local women were undertaken to contextualize the IVR list-based questionnaire and to refine the data collection protocol. The automated IVR questionnaire format was adapted from the Ugandan Demographic and Health Survey (DHS) dietary diversity modules for women and children [54]. First, IVR questions underwent contextualization to identify local sentinel foods commonly eaten in amounts ≥15 g during the wet season in Northern Uganda, as per the Food and Agriculture Organization (FAO) guidelines [30]. For example, foods eaten in very small amounts in sauces such as onions and spices were excluded. The IVR questionnaire was then translated into Lleblango and audio recorded by a female enumerator using a smartphone audio application (Supplementary Table 1). The IVR questionnaire underwent 3 rounds of pretesting and refinement of wording and translation for clarity. The IVR included 3 list-based (yes/no) recall modules: child dietary diversity, women's dietary diversity, and women's handwashing practices. Audio files were uploaded to the engageSPARK platform (v2022), which was programmed with skip logic, invalid response handling, call retries, and reconnects [55]. Project-issued basic phones (Techno T301) were used due to low phone ownership, and network-specific SIM cards were issued to address coverage issues. No airtime was needed since the IVR platform initiated calls.

Lastly, the formative research, including key informant interviews with community leaders and agriculture and health practitioners (*n* = 8), focus group discussions of ≤60 min with between 8 and 10 WRA (*n* = 2 FGDs Apac, *n* = 2 FGDs Kwania), and pretesting of the tool (*n* = 27), identified challenges such as low literacy, unfamiliarity with technology, and socio-cultural behaviors like gender-based violence around use of technology, as also reported in other studies in Northern Uganda [18,56]. Protocols were adapted to include intensive community sensitization, using a female voice in recordings, providing waist bags for secure, discreet phone carrying, scheduling calls during late morning/midday when the woman was likely to be in the privacy of the farm, and using keypad responses.

#### IVR protocol

**FIGURE 2:**
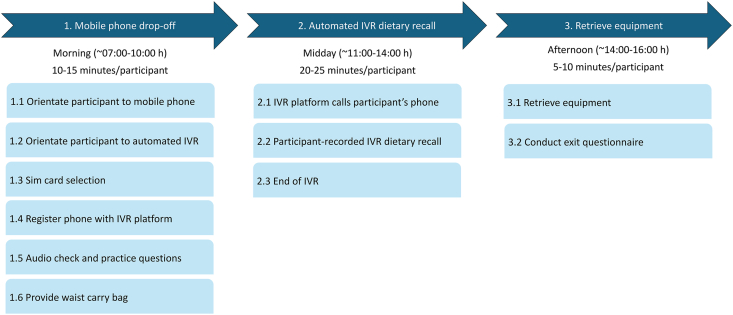
Flowchart of IVR data collection. IVR, interactive voice response.

The IVR protocol is outlined in Figure 2. An enumerator delivered and registered the phone in the morning, provided brief training, and triggered a practice call. The main IVR call was automatically placed 1 h after registration with the platform, with repeated attempts every 15 min if needed. The IVR platform automatically guided the participant through the questions and would reconnect if disconnected due to a poor network. Participants answered questions through keypad responses by pressing “1” for yes, “3” for no, and “8” for do not know. The participant could choose to end the IVR at any time by hanging up and turning off the phone. If the participant failed to give an appropriate response after multiple attempts for the first 2 questions, then the system assumed that the participant did not understand the task and discontinued the call. Equipment was collected in the afternoon, and an exit survey was administered. All IVR procedures followed digital ethical guidelines and privacy legislation.

### Data processing

IVR response data were downloaded from engageSPARK website in Excel. Enumerator-administered questionnaires were cleaned on SurveyCTO before download; all other data were double-entered into Excel. Final cleaning was done in Excel and R. Dietary intake was coded into food groups, and WDDS were calculated for each method (WFR, IVR) using the 10 FAO-defined food groups: *1*) grains, white roots/tubers and plantains, *2*) pulses/legumes, *3*) nuts and seeds, *4*) milk and milk products, *5*) meat, poultry and fish, *6*) eggs, *7*) dark green leafy vegetables (DGLV), *8*) vitamin A-rich fruits/vegetables, *9*) other vegetables, and *10*) other fruits [30]. The percentage of women achieving MDD-W was defined as the percentage who consumed foods from 5 or more of the 10 food groups [30]. WFR data followed standard processing, including calculation of ingredient portion sizes from individual household recipes or average household recipes when individual recipes were not available [48]. It is known that operationalizing the conventional ≥15g cut-off can be challenging [30]; therefore, we also assessed the possible effects of over-reporting of food groups via the IVR method on the outcome indicators and validity [32,35]. Therefore, the IVR method was validated against 2 sets of WFR-generated indicators: one set in which only foods consumed in amounts ≥15 g/d were counted, and the other set in which all the foods consumed were counted (i.e., >0 g/d). Discretionary foods from the Ugandan DHS-8 were coded as unhealthy foods (e.g., fried foods, or foods high in sugar or salt) and unhealthy beverages (e.g., sugar-sweetened tea or soda) [54]. Proportions living below $1.90 per day [57], with severe food insecurity according to household food insecurity experience scores [58], or classified as thin [BMI (in kg/m^2^) < 18.5] or overweight/obese (BMI ≥ 25) were also determined. Administrative data were used to determine the number of network providers per village, network connectivity, and IVR reconnects and call duration.

### Statistical analysis

The primary outcomes were MDD-W, WDDS, and percentage of women consuming unhealthy foods and beverages. Cases with no data for IVR were excluded. If a food was recorded on a participant’s WFR but the weight was missing, then substitute weights were input based on medians calculated from the sample [48]. For the IVR, completing up to the end of the women's dietary intake recall period was required for inclusion (question 65) (Supplementary Table 1).

Multiple statistical tests were used to triangulate validity as recommended [49,59]. Wilcoxon signed-rank and McNemar’s chi-square tests compared IVR with WFR (same day) for WDDS (ordinal) and MDD-W (dichotomous), respectively. Agreement between IVR and WFR was assessed using Weighted Cohen’s kappa for WDDS [49,[59], [60], [61]] and Cohen’s kappa for MDD-W and food groups [49,59,61]. Despite known issues with the kappa (i.e., kappa paradox) [62,63], we have used it herein because it is more appropriate when comparing only 2 methods, including for ordinal variables [60], and to retain comparability with other studies. Kappa values were interpreted as: ≤ 0 poor, 0.01–0.20 slight, 0.21–0.40 fair, 0.41–0.60 moderate, 0.61–0.80 substantial, 0.81–1.00 almost perfect [64]. Food group/subgroup proportions were compared using McNemar’s test, and misreporting was assessed with 2 × 2 tables. WDDS agreement between IVR and WFR was evaluated with Bland–Altman plots and limits of agreement (LOA) [43]. Statistical outputs were interpretated for acceptability to predict dietary diversity indicators according to Lombard et al. (2015) [59]. Socio-demographics of IVR completers compared with noncompleters were compared using Mann-Whitney (continuous) and Fisher Exact (categorical) tests. Analyses were conducted in R (v4.4.1) using “dplyr,” “tidyverse,” “psych,” “ggplot2,” and “BlandAltmanLeh” packages [47], with significance at *P* < 0.05. Reporting follows guidelines for inter-method accuracy [49,63], and agreement studies [[61], [65]].

### Ethical considerations

Ethical approval was obtained from the University of Greenwich Faculty of Engineering and Science Ethics Committee (21.3.8.i.e), Uganda National Council for Science and Technology (A24ES), Clarke International University in Uganda (CIUREC/0066), and London School of Hygiene and Tropical Medicine (26645) institutional review board. Approval for digital data collection platforms (engageSPARK and SurveyCTO) was granted in line with UK data protection laws. Written prior informed consent or thumbprint was obtained from all participants. Modest compensation in kind (i.e., a t-shirt, soap, salt, and oil) was given to all participants for their time.

## Results

### Sample characteristics

A total of 156 women participated in the study. The majority completed the IVR, whereas one-quarter were lost to the study (Figure 1; Table 1), primarily due to poor network, followed by user-related (e.g., difficulty understanding or completing the task) or technological challenges (e.g., flat battery).

**TABLE 1:** Comparison of socio-demographic and mobile phone use characteristics of participants who were included in the analysis (*n* = 116) and those without IVR data (*n* = 40).

	IVR – included in analysis (*n* = 116; 74.4%)		Missing IVR data (*n* = 40; 25.6%)		*P*^1^
	*n* (%)	Median (25th, 75th)	*n* (%)	Median (25th, 75th)	
District					
Apac	62 (53.5)		22 (55.0)		1
Kwania	54 (46.6)		18 (45.0)		
Village					
Road accessibility during the wet season: challenging	46 (39.7)		14 (35.0)		0.70
Close to a large waterbody	58 (50.0)		14 (35.0)		0.14
Household					
Number of household members		5.0 (4.0, 7.0)		5.0 (4.0, 7.5)	0.59
% of households living below $1.90/d (2011 PPP)		29.7 (26.0, 37.5)		29.7 (26.0, 37.5)	0.57
Household severely food insecure	76 (65.5)		29 (72.5)		0.44
Female					
Age (y)		25.0 (22.0, 30.0)		27.5 (20.5, 33.5)	0.29
Married/cohabiting	93 (80.2)		37 (92.5)		0.09
Level of education attainment: none or primary incomplete	87 (75.0)		24 (60.0)		0.10
Can read and write	81 (69.8)		29 (72.5)		0.84
Physiological status: lactating	91 (78.4)		32 (80.0)		0.82
BMI		19.6 (18.5, 21.1)		20.3 (18.9, 21.6)	0.10
Thin (BMI <18.5 kg/m^2^)	33 (28.5)		5 (12.5)		0.05
Telecommunications					
Poor network connectivity	0 (0.0)		12 (30.0)		<0.0001
Both network providers in the village (MTN/Airtel)	77 (66.4)		31 (77.5)		<0.0001
IVR call duration (min)		24.7 (23.4, 26.4)		0.0 (0.0, 2.4)	<0.0001
IVR reconnects ≥1 (for those that started the survey)	14 (12.1)		3 (7.5)		0.56
No functioning mobile phones owned by household	19 (12.2)		7 (17.5)		1
Woman has access to a mobile phone	107 (92.2)		35 (87.5)		0.35
Woman does not own a mobile phone^2^	81 (69.8)		31 (77.5)		0.10
Spouse/partner owns the mobile phone usually accessed^2^	51 (44.0)		25 (62.5)		0.32
Frequent mobile phone use (daily/weekly/monthly)^2^	77 (66.4)		20 (50.0)		0.09
Type of mobile phone accessed: Basic^2^	102 (87.9)		33 (82.5)		1
Good/very good prior experience using a phone	100 (86.2)		24 (60.0)		0.001

IVR, interactive voice response; PPP, purchasing power parity.

1 Mann–Whitney U-test for medians and Fisher's Exact Test for categories.

2 For women who had access to a mobile phone.

Around half of the participants lived in the Apac district and resided near large waterbodies (Table 1). Over one-third faced poor road access during the wet season. Severe food insecurity affected over two-thirds of households, and one-third lived below the poverty line (<$1.90/day). The median age of the women was 26 y and most were married and lactating. Most women had not completed primary school, and close to one-third were illiterate. Additionally, one-quarter was classified as thin. No statistically significant differences were observed in basic descriptive statistics between women who completed the IVR and those lost to the study.

Telecommunications data showed that approximately half of women had variable and a tenth had poor network connectivity. Among those who started and completed the IVR, a tenth were reconnected at least once due to network dropout. Although nearly three-quarters of women did not own a phone, most women had access to a phone owned by another household member (generally their spouse/partner). Of women with access to a phone, most had access to a basic mobile phone. Mobile phone use was infrequent for around one-third of women, and three-quarters had positive prior experience with using a phone. Four telecommunication variables were statistically significant (*P* < 0.05) between IVR completers and noncompleters: noncompleters had poorer network connectivity, a single network provider, shorter call times, and a poor prior mobile phone experience than completers.

### Inter-method agreement for WDDS

**FIGURE 3:**
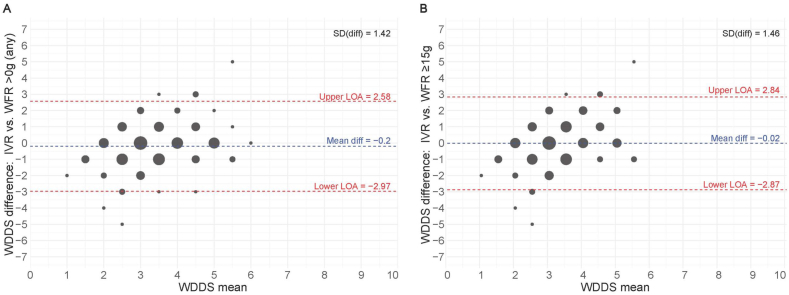
Bland–Altman plots visualizing the difference between mean WDDS of the IVR compared with the WFR at >0 g (any) and ≥15 g minimum requirements for rural Northern Ugandan women (*n* = 116). The size of the bubbles is proportional to the number of participants. Diff, difference; IVR, interactive voice response; LOA, limits of agreement; SD, standard deviation; WDDS, Women’s Dietary Diversity score; WFR, weighed food records.

There were no significant differences in mean WDDS comparing the IVR estimates with those of the WFR (>0 g, ≥15 g; same day) (Table 2). For the WDDS, agreement between the IVR and the WFR was fair for >0 g and ≥15 g, according to the Weighted Cohen’s kappa. Although the Bland–Altman analysis demonstrated no systematic difference in mean score, the LOA were wide, and the plots demonstrate a proportional bias where the differences between methods are proportional to the magnitude of the measurement with the highest disagreement occurring at the lower end of the WDDS (>0 g, ≥15 g) and higher (>0 g only) (Figure 3).

**TABLE 2:** Descriptive statistics and statistical agreement of the WDDS, MDD-W, and discretionary food groups between the IVR compared with WFR for rural Northern Ugandan women (*n* = 116).

	IVR	WFR	
		>0 g (any)	≥15 g^1^
WDDS			
Mean ± SD	3.3 ± 1.5	3.5 ± 1.1	3.3 ± 1.0
Wilcoxon signed-rank test *P* value	-	0.12	0.99
Weighted Cohen’s kappa	-	0.39	0.30
95% CI	-	0.22, 0.55	0.13, 0.47

MDD-W and discretionary food groups			

	*n* (%)^2^		

MDD-W			
≥5/10 food groups	25 (21.6)	20 (17.2)	11 (9.5)^3^
Discretionary food groups			
Unhealthy foods	40 (34.5)	27 (23.3)^4^	-
Sugar-sweetened beverages	38 (32.8)	37 (31.9)	-

CI, confidence interval; IVR, interactive voice response; MDD-W, minimum dietary diversity for women; WDDS, Women’s Dietary Diversity Score; WFR, weighed food record.

1 Given that the ≥15g minimum requirement has been validated as a proxy of higher micronutrient adequacy for the 10 food groups of the WDDS, from which the MDD-W is derived, these food groups were also assessed against this cut-off to explore any effects of misreporting of WDDS food groups on outcomes and validity.

2 McNemar’s chi-squared test demonstrates statistically different proportional pairs.

3 *P* < 0.01

4 *P* < 0.05.

### Inter-method agreement for MDD-W

The percentage of agreement of the MDD-W between the IVR and WFR was high (>80%). According to Cohen’s kappa, agreement for the MDD-W was moderate and fair for >0 g (any) and ≥15 g, respectively (Table 3). The percentage of women achieving MDD-W was not statistically significant between IVR compared with WFR >0 g (any); however, the MDD-W was statistically significantly higher for IVR compared with WFR ≥15 g (Table 2).

**TABLE 3:** Inter-method agreement for estimating MDD-W and discretionary food groups between the IVR and the WFR for rural Northern Ugandan women (*n*=116).

	IVR		Agreement statistics		
MDD-W					
	<5 Food groups	≥5 Food groups	% Agreement	Cohen’s kappa	95% CI
	*n* (%)				
WFR >0g (any)					
<5 Food groups	84 (72.4)	12 (10.3)^3^	83.6	0.48	0.28, 0.68
≥5 Food groups	7 (6.0)^2^	13 (11.2)			
WFR ≥15g^1^					
<5 Food groups	88 (75.9)	17 (14.7)^3^	82.8	0.36	0.15, 0.57
≥5 Food groups	3 (2.6)^2^	8 (6.9)			

Discretionary food groups					
	None	Any	% Agreement	Cohen’s kappa	95% CI
	*n* (%)				
	Unhealthy foods				
WFR >0 g (any)					
<0 g (none)	69 (59.5)	20^3^ (17.2)	76.7	0.44	0.27, 0.61
>0 g (any)	7^2^ (6)	20 (17.2)			
	Sugar-sweetened beverages				
WFR >0 g (any)					
<0 g (none)	64 (55.2)	15^3^ (12.9)	75.0	0.43	0.25, 0.60
>0 g (any)	14^2^ (12.1)	23 (19.8)			

CI, confidence interval; IVR, interactive voice response; MDD-W, minimum dietary diversity for women; WDDS, Women’s Dietary Diversity Score; WFR, weighed food record.

1 Given that the ≥15 g minimum requirement has been validated as a proxy of higher micronutrient adequacy for the 10 food groups of the WDDS, from which the MDD-W is derived, these food groups were also assessed against this cut-off to explore any effects of misreporting of WDDS food groups on outcomes and validity.

2 False negative finding (type II error).

3 False positive finding (type I error).

### Inter-method agreement for unhealthy foods and beverages

The proportion of women consuming sugar-sweetened beverages was not statistically different between IVR compared with WFR, but it was for unhealthy foods (Table 2). Agreement for unhealthy foods and beverages, comparing IVR with WFR, was 75%, with moderate Cohen’s kappa values (Table 3).

### Inter-method agreement for food groups

Food group agreement ranged from >70% to >95%, highest for fish/meat/poultry and DGLVs (substantial), and dairy and sugar-sweetened beverages (moderate) (Supplementary Tables 2 and 3). Agreement was lowest for pulses (fair). Most false positives were for vitamin A-rich fruits/vegetables (around 1 quarter) and unhealthy foods (< 1 quarter), whereas false negatives were more common for pulses (1 quarter), grains/white roots (around 1 quarter), and other vegetables (around 1 quarter).

### Acceptability

Most women reported they had a positive IVR experience, and >95% were willing to use IVR dietary reporting in future studies. Qualitative feedback was mostly positive, with 1 participant reporting that the IVR was “Easy to use and answer” (ID = 100) and another stating “Excited since [I] had never used a phone before” (ID = 52). Some reported challenges, including participants reporting fear due to limited phone knowledge (ID = 87), lack of confidence answering questions (ID = 70), and concerns about call length and repeated questions (ID = 97).

## Discussion

Our results outline the validity of an automated participant-recorded list-based recall of food groups consumed during the previous 24 h using push-button response (yes/no) via IVR on basic mobile phones for assessing population-level MDD-W, WDDS, and percentage consuming unhealthy foods and beverages among rural women (18–49 y) during the wet season in Northern Uganda, using criteria specified by Lombard et al. (2015) [59]. Here we demonstrate the feasibility of IVR for monitoring women's dietary quality in a challenging context, including heavy rains during the wet season (i.e., interruptions to network connectivity), risk of gender-based violence around the use of mobile phones, and low literacy and limited phone experience among rural women, supporting the robustness and usefulness of this method for collecting automated dietary data from population groups with high food insecurity in a rural resource-scarce settings. Most women successfully completed the IVR, with completion associated with better network coverage and prior positive mobile phone experience. We found no significant differences comparing the IVR estimated mean WDDS and the proportion achieving MDD-W with those estimated from the gold standard observed WFR, indicating the potential for using this automated mobile-phone-based method for monitoring key international dietary quality indicators.

This study is the first to assess the validity of automated IVR for collecting data to assess dietary diversity using basic mobile phones among rural women in a sub-Saharan context, making direct comparisons with the literature difficult. We found a high percentage agreement of the IVR for estimating MDD-W against the WFR (84.4%; same day), similar to a study validating the ability of enumerator-administered surveys via telephone compared with in-person surveys to estimate MDD-W in Kenya (74.4%; different day) [20]; however, our level of agreement may be slightly higher due to the same day comparison compared with their different day comparisons. The ability of our IVR to estimate mean WDDS (weighted *kappa* = 0.39 ) and MDD-W (*kappa* = 0.48) is also similar to a study validating the ability of enumerator-administered list-based recalls of food groups consumed in the previous 24 h to estimate mean WDDS (weighted *kappa* = 0.47) and MDD-W (*kappa* = 0.51) against observed WFRs in Cambodia, Ethiopia, and Zambia [36]. Although these studies are not directly comparable, these results indicate that our findings are at least consistent with other validation studies of MDD-W and WDDS in LMICs using enumerator-administered mobile phone surveys and list-based recalls of food groups consumed in the previous 24 h.

Although there is not a biologically important mean systematic bias, the Bland–Altman plots indicate the direction of the bias changes in proportion to the magnitude of the measurement, and the LOA is wide. The latter result supports the wider literature that the MDD-W and WDDS should only be used at the population level, as the large random error will attenuate associations between dietary diversity indicators and health outcomes [[31], [32], [33],^37^]. The wide LOA indicates that IVR will overestimate the MDD-W prevalence estimate compared with the WFR, in populations with a median WDDS well below 5 food groups, as was observed in our study. Most importantly, the results of the IVR to estimate the exact WDDS and MDD-W at the individual level should not be overstated. Although the Cohen’s kappa values fall within the “acceptable” range for dietary validation studies [59] and are consistent with expectations for a sample of this size [44], the agreement is fair to moderate, supporting the wider literature on the inherent weaknesses of self-reported dietary data [49,50]. Thus, our study indicates that the agreement is sufficient for the IVR to estimate cross-sectional population-level dietary diversity indicators, but caution should be taken if assessing associations between dietary diversity and health outcomes.

In this study, the IVR accurately identified women consuming nutrient-rich foods like fish/meat/poultry, DGLVs, and dairy compared to WFRs (Supplementary Tables 2 and 3). However, the IVR over-reported vitamin A-rich fruits and vegetables and unhealthy foods, and under-reported white roots/tubers, pulses, and other vegetables compared with WFR. It is known that misreporting is common with self-reported dietary data due to factors such as memory lapses, social bias, telescoping, and aspirational reporting [48,50], especially for list-based recalls where there is the requirement for criterion foods listed, for each food group, to be asked in the questionnaire [35,37,66]. In our study, some misreporting may have stemmed from confusion about what qualifies as an orange-fleshed sweet potato, a distinction that usually requires trained enumerators and probing [30]. The order of questions may have contributed, as the consumption of vitamin A-rich vegetables was asked before white roots/tubers (Supplementary Table 1). Future IVR-administered recalls should carefully test question order and wording for these food groups to improve accuracy.

Our findings indicate that food group misreporting was higher when using a ≥15 g minimum compared with a >0 g (any) threshold, underscoring the challenges of implementing quantitative cutoffs in list-based food group recalls [37]. Although the literature indicates that both thresholds serve as proxies for micronutrient adequacy at the population level, the ≥15 g cut-off has a stronger association with micronutrient adequacy than the >0 g cut-off in a number of studies [31,32,35,37]. The use of a ≥15 g cut-off is challenging, but efforts should continue to develop strategies to exclude foods eaten in very small amounts in participant-recorded methods [30]. In future IVR studies, particular attention should focus on identifying sentinel foods for inclusion on the IVR questionnaire that are consistently eaten in amounts ≥15 g/d. In rural Northern Uganda, we found that eggs and small protein foods (i.e., insects) were eaten infrequently and in predominantly small amounts, indicating that these foods should continue to be disaggregated from other animal source foods, to prevent over-inflation of the fish/meat/poultry food group, as per the current FAO guidelines [30]. Although tomatoes and nuts/seeds were included in sauces, okra leaves, and dried fish were sometimes eaten in small amounts, we found that overall, these foods were eaten in amounts ≥15 g, indicating they can remain on the sentinel food list for this context.

Consistent with other mobile phone-based studies in LMICs [6,7,25], we found high acceptability for IVR, with most women reporting their willingness to use mobile phones for future dietary reporting. Prior positive experience with basic mobile phones was common and significantly associated with successful IVR completion. Most participant loss was due to poor network connectivity, worsened by heavy rain, yet completion rates remained high (>70%), like IVR validation studies in high-income countries [28] and other phone-based surveys in sub-Saharan Africa [14,20]. Tailoring SIM cards to the best local network and using automatic reconnect features improved completion, while providing waist bags helped women keep phones within hearing range during daily activities. Smartphone ownership was rare, confirming the suitability of basic mobile phones in this context. Careful contextualization, translation, and simplification of IVR questions, plus community sensitization and use of a female-voiced audio recording, were key for community acceptability and participant safety [18,56], supporting the use of mobile phone-based dietary data collection in gender-sensitive settings.

By providing mobile phones and short training sessions, we enabled participation irrespective of women's literacy and phone ownership status, negating noncoverage bias, which can result in misidentification of population-level dietary quality rates by 25% in sub-Saharan African phone-based studies [20]. Although providing basic mobile phones incurred a modest upfront cost (∼$10–15/phone), many women had access to a basic phone via a household member, suggesting future studies may be able to use existing devices. However, we found that individual training was critical for women new to mobile phones, highlighting the importance of tailored support to foster equitable participation in settings with high gender inequalities [13] or higher asset ownership among older women [14,20]. As individual and household phone ownership continues to expand across LMICs [16,24], it is anticipated that the cost-effectiveness of automated participant-recorded approaches will increase [8,13,14]. Yet the need for participant training among low-literate participants suggests that IVR may be best suited to high-frequency monitoring of sentinel groups, with IVR positioned to complement traditional monitoring systems, particularly during wet-season food shortages or other times when enumerator mobility is restricted [7,10,14,15]. The real-time insights of high-frequency data could also track seasonal and environmental impacts on dietary quality, addressing the chronic scarcity of longitudinal data in LMICs [11,34]. Studies validating the IVR for longitudinal tracking of dietary diversity indicators are warranted in this and other low-resource contexts.

This study had several limitations. It was a cross-sectional study conducted in 1 season, limiting the assessment of seasonal differences in agreement between IVR and WFR dietary diversity indicator estimates. Three villages were excluded because of flooding. Like all dietary recall methods, inherent weaknesses such as omissions and misreporting due to memory errors or social bias can attenuate accuracy [49,50]. The presence of the enumerator who weighed all foods and beverages consumed over the day may have resulted in participants altering their eating habits, and “primed” the respondent to complete the IVR and remember what they ate when they were asked on the following day [48]. Although the study’s extended interest in women's activities and handwashing practices might have slightly reduced the bias, the IVR response/completion rates and level of agreement for dietary diversity indicators might be lower in a general survey than in the current validation study. Due to resource constraints, repeated measures were not possible, limiting the assessment of reliability. Although our sample size was comparable to other dietary validation studies [41], the wide CIs indicate that larger sample sizes may be required in future studies to narrow the CI around the systematic and proportional bias [63] and allow for subgroup analysis [42]. This study was part of a broader research project, which may have influenced participation and reporting. Respondent fatigue may have contributed to food group under-reporting, given that the women's dietary recall came after the child’s dietary recall, in the IVR questionnaire. A shorter IVR questionnaire, which only asked about the women's diet, may have improved accuracy [21]. Although WDDS and MDD-W capture the diversity dimension of dietary quality, serving as proxies for micronutrient adequacy, the unhealthy food and beverage consumption only partially addresses the moderation dimension; thus, future studies would be strengthened by assessing additional dimensions of dietary quality [34,67]. Future IVR studies could assess the use of the standardized Diet Quality Questionnaire, recently adapted for Uganda and many other countries (www.dietquality.org), from which multiple dietary quality indicators spanning these dimensions can be derived, enhancing cross-country comparability and alignment with international healthy diet monitoring efforts (www.healthydietsmonitoring.org) [68,69].

Strengths of this study included the use of observed WFRs as the criterion method [48,49], and multiple statistical tests to triangulate validity [49,59,61]. Formative research informed contextualization of the IVR questionnaire and protocol refinement, and the tool was validated during the wet season with rural, low-literate women in a patriarchal context, demonstrating robustness under challenging conditions [18,56]. The sample included a diverse mix of agricultural-dependent households (e.g., fisheries, crop, pastoralist) and all reproductive physiological stages (lactating, pregnant, nonpregnant/nonlactating), supporting applicability for WRA in a rural environment with diverse livelihoods. Future research needs include validation of the IVR method for diverse contexts, including other vulnerable groups, such as adolescent girls, and for remote longitudinal monitoring.

In conclusion, this is the first study to assess the validity of a participant-recorded, list-based recall of food groups consumed in the previous 24 h using IVR with push-button response on basic mobile phones among low-literate rural women in sub-Saharan Africa during the wet season. With short participant training, the voice-based survey and keypad response bypassed literacy barriers and proved feasible in a resource-limited setting, with Cohen’s kappa demonstrating fair-to-moderate agreement compared with observed WFRs. In contexts where resource-intensive dietary surveys are constrained, IVR may provide a pragmatic alternative for population-level estimation of key international dietary quality indicators. This approach offers a low-cost, automated solution for identifying group differences and assessing trends across time. However, trade-offs between simplicity and accuracy, combined with the need for training of low-literate participants, indicate that this IVR method may be best suited to high-frequency monitoring of sentinel groups, although further longitudinal validation studies are required. As risks from climate change, conflict, and health crises increase, IVR-based dietary reporting can support more equitable and timely nutrition monitoring. Further refinement and validation across additional contexts could enhance accuracy, extending the application of IVR across other low-resource contexts.

## Author contributions

The authors’ responsibilities were as follows –KW, EF, JN, LO: conceptualization; LO: data curation, formal analysis, investigation, visualization, software, and writing—original draft; KW, EF, JN: funding acquisition; LO, EF, KW, JN, PO: methodology; LO, KW, JN: project administration; LO, KW, JN, PO: resources; KW, EF, PD-S: supervision; LO, EF: validation; LO, EF, KW, PD-S, JN, PO: writing—review and editing; and all authors: read and approved the manuscript.

## Data availability

Data described in the manuscript, code book, and analytic code are publicly and freely available without restriction. Data are available at Harvard Dataverse: "Replication Data for: validation of interactive voice response (IVR) for collecting dietary data among low-literate women in Northern Uganda 2022": https://doi.org/10.7910/DVN/SPKOQB. R script for analysis is available on github: https://github.com/l-c-omeara/ivr-validation.git.

## Declaration of Generative AI and AI-assisted technologies in the writing process

During the preparation of this work, the main author (LO) used ChatGPT to refine R code, and Perplexity for grammar, spelling, and word count reduction. After using these tools, all authors reviewed and edited the content as needed and take full responsibility for the content of the published article.

## Funding

This study was financially supported by an Innovative Methods and Metrics for Agriculture and Nutrition Actions (IMMANA) grant, funded by UK AID (IMMANA 3.01). LO was a PhD candidate supported by Research England through the Food and Nutrition Security Initiative (50.18-E3) at the University of Greenwich. The funders had no influence on the research findings or reporting of this study.

## Conflict of interest

The authors have no conflicts of interest to declare.
